# Interdisciplinarity of secondary osteons: relevance of bioarchaeological and palaeontological research in biomedical cortical bone histology studies

**DOI:** 10.1016/j.bonr.2025.101893

**Published:** 2025-12-05

**Authors:** Justyna J. Miszkiewicz, Karen M. Cooke, Holly E. Reid, Julien Louys

**Affiliations:** a School of Social Science, University of Queensland, Brisbane, Australia; b Vertebrate Development, Ecology and Evolution, Naturalis Biodiversity Center, Leiden, the Netherlands; c Australian Research Centre for Human Evolution, Griffith University, Brisbane, Australia

**Keywords:** Histomorphometry, Histomorphology, Haversian systems, Interdisciplinarity

## Abstract

Secondary osteons are fundamental products of bone metabolic processes. They accumulate, crowd, and superimpose over themselves over the lifespan of the individual and capture significant biological, environmental, and even social information. They are crucial in biomedical research as a reflection of bone health and disease. Because bone preserves after death, secondary osteons offer a window into past human and animal lives. They are arguably one of very few biological traits that have been examined across multiple disciplines that work with modern and ancient samples. Here, we review articles indexed in PubMed that examine secondary osteons but span beyond biomedicine, such as palaeontology and bioarchaeology. We aim to identify commonalities and differences across these disciplines to highlight potential for exchange of existing complementary data and future collaborative avenues. We find that 9 % of articles reporting new secondary osteon data (622 obtained) represent archaeological or fossil material. The key shared positive outcome across these disciplines has been data and histology images that provide insights into age, sex, behaviour, species discrimination, and anatomical variation. The main limitations of using ancient samples are the unknown and thus estimated demographic information of human and animal remains studied, and artifacts of taphonomy and bioerosion seen in bone histology that originate from post-mortem, burial, and diagenetic processes, which are not present in biomedical samples. We conclude that a histological analysis of secondary osteons can be a versatile tool in different fields of bone research and encourage transdisciplinary collaboration to better improve our knowledge of bone remodelling processes.

## Highlights

• Secondary osteons can be examined in ancient and modern samples.
• PubMed articles examining secondary osteons in ancient bone are reviewed.
• We find biomedically relevant aims, such as variation with age, sex, biomechanics.
• Poor preservation and estimated demographics limit ancient bone histology.
• Interdisciplinary exchange of ancient and modern secondary osteon data is urged.

## 1 Introduction

**Fig. 1:**
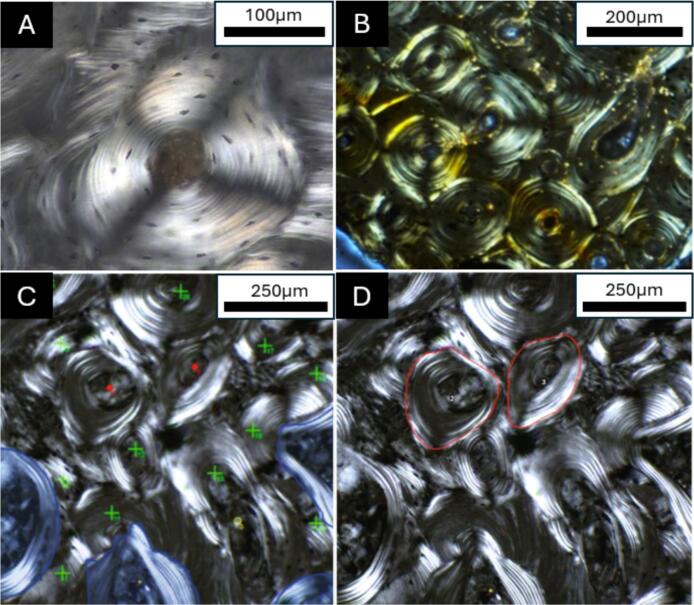
A selection of secondary osteon examples in cortical bone viewed under polarised light in bones originating from different time periods, all showing excellent preservation that allows for histomorphometric and histomorphological analyses of bone remodelling. A shows a secondary osteon with visible lamellae and osteocyte lacunae in the femur of an archaeological human dated to the medieval period (11th–16th century) in England, UK. B shows a series of secondary osteons with a range of collagen fibre orientations in the rib of a fossil hippopotamus from Cyprus dated to the Pleistocene. C-D show intact (red dots in C, defined as secondary osteons with complete/intact visible cement lines that are not interrupted by another secondary osteon) and fragmentary (green crosshairs in C; defined as partially visible secondary osteons where the entirety of the cement line is not visible due to a separate remodelling event partially resorbing the preexisting secondary osteon) secondary osteons that can be counted and measured (e.g. area indicated in D with a red trace) in a femur from a modern human donor (Townsville, Australia). All images are reproduced based on open access permissions: image A CC-BY-4.0 from Miszkiewicz et al., 2022, image B CC-BY-4.0 from Miszkiewicz et al., 2023, images C-D CC BY-NC 4.0 from Pedersen et al., 2024.

A secondary osteon is a fundamental structural unit of cortical bone, and it is formed by the Basic Multicellular Unit (BMU) shaped like a cutting cone (Chang and Liu, 2022; Lassen et al., 2017). In ground histology sections taken in a transverse plane, secondary osteons appear as roughly circular structures that have a central pore for blood supply (Haversian canal) surrounded by lamellae composed of collagen fibres and hydroxyapatite crystals (Fig. 1). The boundary of a secondary osteon is known as a cement line, which is thought to be highly mineralised (in relation to the osteon) (Cantamessa et al., 2025; Delaisse et al., 2021; Skedros et al., 2005). There is also an outer margin of the space resorbed by osteoclasts signifying their reversal (reversal line) (Skedros et al., 2005). The lamellae are deposited by osteoblasts in layers formed of osteoid which becomes gradually calcified (Smith et al., 2012). As such, secondary osteons form as a result of intricate and focal remodelling processes where coupling between osteoclasts and osteoblasts ensures that bone is resorbed and deposited on the same surface and where remodelling is needed (Sims and Martin, 2020; Sims, 2024). Remodelling is fundamental to bone health and strength, as it ensures calcium reservoirs are replenished and microdamage is repaired, making secondary osteons some of the most important microscopic features of the human skeleton (Kenkre and Bassett, 2018). While modelling is also important, as it shapes bone during growth and development, it removes bone from one surface and adds bone tissue to another without the creation of secondary osteons (Allen and Burr, 2019a, Allen and Burr, 2019b).

Histological analysis of secondary osteons can be static or dynamic, and can take the form of histomorphometric or histomorphological approaches (Chavassieux and Chapurlat, 2022). Static histology generates fixed data from samples or images analysing structures and components of secondary osteons such as lamellae and osteocyte and their lacunar morphology. This method works on samples that are stained and unstained, decalcified and undecalcified and yields data that can be used in reconstructing bone remodelling or BMU activity retrospectively from measurements or qualitative descriptions. Dynamic histology generates real-time data to capture sequential changes in bone microstructure (Varela and Jolette, 2018). For example, it relies on fluorescent labels administered in vivo and over a specified timeframe so that bone resorption and formation can be measured from within secondary osteons or lamellar bone (e.g. Bromage et al., 2009). The timing of tetracycline label injection can also have forensic significance when identifying human remains (Stout, 2009). Both static and dynamic cortical bone histology can allow examination of secondary osteons quantitatively or qualitatively, although dynamic histology naturally favours quantification over qualitative descriptions.

Histomorphometric and histomorphological methods applied to secondary osteon examination can explore different parameters depending on the research question. Using static techniques, secondary osteons can be assessed based on their number or density within a histological region of interest, which tells us how much bone has been produced/remodelled per given area (Frost, 1987). It is a good measure of localised remodelling that can be linked to factors or known contexts such as mechanical load or nutrition (Gocha and Agnew, 2016; Paine and Brenton, 2006). Another measure is based on geometric properties such as area, diameter, width, or circularity, which reflect the size of BMU at different stages of one remodelling cycle (Cole et al., 2025). Dynamic histomorphometry is more concerned with quantifying measures such as bone formation rate or mineral apposition rate, typically over a known time or developmental period (Allen and Burr, 2019a, Allen and Burr, 2019b).

Variation in morphology and morphometry of secondary osteons in cortical bone has been extensively studied in biomedical research, particularly in understanding bone metabolic diseases and cortical bone adaptation to mechanical load and implants in surgical contexts (Bernhard et al., 2013; Maeda et al., 2018; Hofmann et al., 2006; van Oers et al., 2008a). It is also worth noting that although secondary osteons do not form through a cutting cone in trabeculae, the remodelling process there excavates ‘trenches’ leading to the creation of hemi-osteons, which can be useful in comparing remodelling activity between cortical and trabecular bone (van Oers et al., 2008b). Other than medical fields, the analysis of secondary osteons appears also in disciplines that deal with ancient animals and humans, such as palaeontology and bioarchaeology (where such investigations are broadly termed ‘palaeohistology’) (de Ricqlès, 2011; Houssaye, 2014; Chinsamy, 2023; Stout and Crowder, 2025; Miszkiewicz and Mahoney, 2017). These can directly overlap with medical subjects, such as in cases of studying ancient behaviour and bone adaptation to different environmental stimuli. Given this interdisciplinary overlap, it is worth investigating to what extent secondary osteon analysis on ancient samples can make contributions to the biomedical space, potentially expanding our current knowledge of bone biology from bone sources that are not typically considered by clinical teams. Here, we review the interdisciplinary nature of secondary osteons by identifying bioarchaeological and palaeontological research of these microscopic structures published in peer reviewed articles indexed by PubMed, the free and searchable bibliographic database of biomedical literature. We use these to explore what commonalities and irreconcilable differences may exist across bone disciplines that work with modern and ancient samples to identify avenues and make recommendations for practitioners in both areas to highlight potential for exchange of existing complementary data and future collaborative avenues.

## 2 Methods

This literature review-based study follows standard methods when searching scientific literature databases using keywords (Anders and Evans, 2010; Nourbakhsh et al., 2012). We focused on PubMed using keywords based on secondary osteon histological parameters outlined in the Introduction, entered without quotation marks to not limit searches to exact phrasing (Table 1). The secondary osteon parameters were grouped into four main types within two major categories: static histology, comprising density, geometric properties, collagen fibre orientation measures; and dynamic histology, comprising parameters measured from labelled samples (Table 1). Where the parameter did not include ‘osteon’ in its name, the term ‘osteon’ was added for clarity. This was the case for osteocyte lacunar density, and the dynamic histology measures of bone formation rate, mineral apposition rate, and activation frequency.

**Table 1:** Grouping of bone histological parameters for the purpose of literature search and based on common characteristics within these techniques.

Histological grouping based on methods and parameter determination	Keywords based on parameters specific to secondary osteons
Static histology	
Density (histomorphometry)	Osteon population density
	Osteocyte lacunar density, osteon
Geometric properties (histomorphometry)	Osteon area (including Haversian canal)
	Osteon circularity
	Osteon wall thickness
Osteon morphotypes (histomorphology)	Collagen fibre orientation, osteon

Dynamic histology	
Fluorescent labelling (histomorphometry)	Bone formation rate, osteon
	Mineral apposition rate, osteon
	Activation frequency, osteon

The search spans the database entries available to 25 September 2025. Only peer reviewed original articles in English were included, and excluded theses, preprints, conference abstracts, books, and book chapters. Following a search using a keyword, we recorded the total number of articles returned and downloaded the entire citation record (see Supplementary Material). Each reference was reviewed in full to ensure it met these criteria and that it reports results of analyses on secondary osteons using one or more of the specified histological parameters described in Table 1. The resulting record for each parameter was then screened to identify publications that use archaeological and/or palaeontological bone samples. Any article with multiple histological variables was tallied individually per parameter but merged in the Results tables. From each article we extracted information about bone examined, sample size, the nature of the material (fossil/archaeological), its historical, geological or depositional age, provenance, and research question investigated. Our analysis was qualitative and focussed on three areas for critical review: the choice of parameters investigated, bone type and research design, and technical points.

## 3 Results and discussion

Using the histological terminology relating to secondary osteons, we recorded 622 articles that spanned anatomy, (bio)medicine, biomechanics, orthopaedic surgery and engineering, dentistry, veterinary science, forensic science, zooarchaeology, bioarchaeology, and palaeontology (see Supplemental Material). Following screening for studies on ancient samples, we identified 25 peer reviewed articles that reported the use of archaeological human remains (Table 2, Table 3) and 11 that reported the use of fossil material (Table 2, Table 4) (these counts include articles not repeated across different histological parameters).

**Table 2:** The number of articles returned during the PubMed search per given keyword as a total output, following a screen of references per parameter, and then following a manual review of each article.

PubMed search	Total number of articles returned	Number of articles after screen for bioarchaeological and palaeontological literature per PubMed output	Number of articles after screen for bioarchaeological and palaeontological literature per manual review
Osteon population density	72	7 (9.7 %)	26
Osteocyte lacunar density, osteon	25	0	2
Osteon area	90	19 (21.1 %)	12
Haversian canal area	192	20 (10.42 %)	10
Osteon circularity	14	3 (21.43 %)	3
Osteon wall thickness	14	2 (14.29 %)	1
Collagen fibre orientation, osteon	53	1 (3.8 %)	0
Bone formation rate, osteon	91	2 (2.2 %)	1
Mineral apposition rate, osteon	28	0	0
Activation frequency, osteon	43	1 (2.3 %)	1
Totals (includes articles repeated in different parameters)	622	55	56

**Table 3:** Results from our search identifying archaeological specimens reported in articles in journals indexed by PubMed.

Reference (alphabetical)	Bone	N	Parameters	Subject, age, provenance	Research question
Burr et al., 1990	Human femur	55	Osteon densities, osteon wall thickness	14th–19th century Pecos Indians, New Mexico (USA)	Bone histological change in ancient and modern populations
Caccia et al., 2016	Human femur, skull fragments (bone not specified)	31	Osteon densities	2nd-4th centuries Roman graveyards, 17th century mass grave in Milan, 1st century BC Roman burial in Brescia (Italy)	Histological structure of bone in juveniles and adolescents
Cooke et al., 2022; Miszkiewicz, 2016; Miszkiewicz and Mahoney, 2016; Pitfield et al., 2017, Pitfield et al., 2019a, Pitfield et al., 2019b	Human femur, humerus, rib	50–450	Osteon densities, Haversian canal and osteon area, osteocyte lacunar densities	11th–16th centuries medieval England, Canterbury (UK)	Bone remodelling differences between social groups, ages, sexes; occurrence of osteon variants
Cummaudo et al., 2018	Human humerus, ulna, radius, clavicle, femur, tibia, fibula, metacarpal, metatarsal, glabella, frontal bone, parietal, occipital, temporal bone, scapula, sternum, rib, iliac crest, os coxa, sesamoid, patella, mandible, cervical vertebra	1	Osteon densities	Medieval San Martino di Serravalle's Church (Italy)	Variability of histological structure in different parts of the human skeleton
Cummaudo et al., 2019	Human and pig humerus, radius, ulna, femur, tibia, fibula	1	Osteocyte lacunar density, osteon circularity and area	Medieval, details not reported	Discrimination of species (human vs. pig) based on bone histology
de Gruchy et al., 2024	Human femur	20	Osteon densities	1833–1916 Cemetery of St. Anne, Brussels (Belgium)	Intra-section variation in bone remodelling with isotopic signatures
Iwaniec et al., 1998	Human femur	94	Osteon densities	Inuit and Pueblo agriculturists, details not reported	Accuracy of osteon density calculations from different sampling bone regions
Karydi et al., 2022	Human femur, rib	14	Osteon densities, circularity	1839 and 1857 St. Peter's burial ground in Blackburn (UK)	Age estimation from bone histology
Lagacé et al., 2020	Human and animal femur, tibia, humerus	5 human, 2 deer	Haversian canal area, osteon area, osteon densities, osteon circularity	Medieval Beaulieu and Notre-Dame (France)	Species differentiation based on bone histology
Miszkiewicz et al., 2020	Human femur	1	Haversian canal area	Metal Period (∼2000 BP) Nagsabaran, Luzon Island, Philippines	Sided analysis of bone histological variation in relation to disuse osteoporosis and hip joint ankylosis
Miszkiewicz et al., 2022	Human femur	69	Haversian canal area and osteon area, osteon densities	440–150 BP Taumako (Solomon Islands)	Examination of bone porosity linked to experiences of osteoporosis with a sex and age comparison
Mulhern and van Gerven, 1997; Mulhern, 2000	Human rib, femur	43–80	Osteon number, osteon and Haversian canal area, activation frequency, bone formation rate	550–1450 medieval Kulubnarti (Sudanese Nubia)	Bone remodelling and histomorphometric variation with age and sex
Pfeiffer, 1998; Pfeiffer et al., 2006	Human femur, rib	41–84	Osteon and Haversian canal area	Holocene (Later Stone Age) foragers (South Africa), 1729–1857 Spitalfields (UK), 1827–1873 St. Thomas (Canada)	Variation in bone histomorphometry with behaviour
Pfeiffer et al., 2019	Human rib	54	Osteon densities	Later Stone Age, Greater Cape Floristic Region (South Africa)	Past human health, adaptations, diet and lifespan inferred from bone histology
Pitfield et al., 2019a	Human humerus, rib	175	Haversian canal area	Medieval Canterbury, York, Newcastle (UK)	Physical activity and bone histomorphometry in children
Raguin and Drapeau, 2020	Human humerus, femur	23	Osteon densities, osteon and Haversian canal area	1771-1860 St. Matthew historical cemetery, Quebec City (Canada)	Formation of double zonal osteons and links with past load inferred from bone cross-sectional geometric properties
Thompson and Gunness-Hey, 1981	Human femur	258	Osteon densities and area, Haversian canal area	19th century Inuit from St. Lawrence Island, Baffin Island, Southampton Island, and 700 B.C. to 17th AD Kodiak Island (Canada)	Comparison of bone remodelling dynamics between archaeological Inuit and modern US populations
Walker et al., 2023	Human femur	17	Osteon densities, Haversian canal area and osteon area	906–1523 cal. BC Mán Bạc, (Vietnam)	Bone remodelling variation with ancestry
Woide et al., 2010	Human fibula, tibia, humerus	4	Osteon densities and geometric properties	c. 1550–1070 BC Egyptian mummies, Thebes-West (Egypt)	Laser microdissection of osteon bone for ancient DNA

**Table 4:** Results from our search identifying palaeontological specimens reported in articles in journals indexed by PubMed.

Reference (alphabetical)	Bone	N	Parameters	Subject, age, provenance	Research question
Bergqvist et al., 2019	Osteoderm	15	Osteon densities	*Riostegotherium yanei* and *Dasypus novemcinctus*, Itaboraí Basin (Brazil)	Histological organisation of osteoderms
de Andrade and Sayão, 2014	Femur, tibia	2	Osteon size and densities	Dyrosaurid, Maria Farinha Formation (Brazil)	Biology and lifestyle of dyrosaurids
de Farias et al., 2024	Rib, humerus, radius, fibula, tibia, femur	9	Osteon densities	*Decuriasuchus quartacolonia*, Triassic (Brazil)	Inferring growth patterns and ontogeny from bone histology
Downs and Donoghue, 2009	Dermal skeleton of the antiarch placoderm	13	Osteon densities	*Bothriolepis canadensis*, Frasnian-age exposures of the Escuminac Formation, Quebec (Canada)	Description of the dermal skeleton of the antiarch placoderm
Huttenlocker and Botha-Brink, 2014	Radius, tibia, ulna, humerus, femur, fibula	11 genera	Osteon size and densities, but mostly primary	Therocephalians, Middle Permian through Middle Triassic of the Karoo Basin (South Africa)	Phylogenetic survey of limb bone histology and growth patterns
Martinez-Maza et al., 2014	3 humeri, 4 femora, 6 tibiae, 10 metatarsals, and 7 metacarpals, metapodials	26	Osteon densities	*Hipparion concudense*, upper Miocene localities of Segovia province and Concud (Spain)	Growth patterns and life history traits in Hipparion (extinct equine genus)
Perillo and Sander, 2024	Indeterminate cortical fragments	6	Osteon densities	*Shonisaurus sikanniensis*, Shastasauridae gen. et sp. indet., Tetrapoda indet. Late Triassic localities across Europe: presumable ichthyosaur from Lilstock (England) Autun (France), Bonenburg (Germany), and Canada	Resolving taxonomic questions relating to unidentified fossils from European localities
Ponce et al., 2023	Femur, rib, osteoderm, fibula, humerus	2	Osteon densities	*Fasolasuchus tenax* (Argentina) and *Prestosuchus chiniquensis* (Brazil)	Palaeobiological inferences, growth rate, and skeletal maturity
Sayão et al., 2016	Rib, ulna	1	Osteon densities	Crocodyliform from Early Cretaceous, Crato Formation, Ceará (Brazil)	Growth pattern inferred from bone histology
Skutschas and Stein, 2015	Humerus, femur	9	Osteon densities	*Kokartus honorarius*, Middle Jurassic (Kyrgyzstan)	Histological analysis of long bones
Sombathy et al., 2025	Tibia, femur, rib, osteoderm	4	Osteon densities	*Ceratosaurus* non-avian theropod dinosaur, Upper Jurassic Morrison Formation (North America)	Histological and growth model estimates of life history in Ceratosauria

The largest proportion of bioarchaeological or palaeontological literature represented cortical bone histological parameters that measured osteon densities, followed by geometric properties of secondary osteons and Haversian canals. Most articles are repeated across different parameters because they typically used a suite of histological variables. For the parameter of osteocyte lacunar density, our PubMed search did not yield any articles with archaeological or fossil origin upon first investigation, but once the articles for other parameters were screened, we identified Miszkiewicz (2016) and Cummaudo et al. (2019), who did examine osteocyte lacunae in human and animal samples.

### 3.1 Secondary osteon parameters

Bioarchaeologists and palaeontologists have used measures of osteon densities and geometric properties of osteons and Haversian canals to calculate the number of osteon generations per mm^2^ to determine chronological age (Karydi et al., 2022), explore bone histological change with age (e.g. Mulhern and van Gerven, 1997; Mulhern, 2000; Caccia et al., 2016; Pitfield et al., 2017), and to relate bone remodelling to mechanical stimulation or behaviour within and between temporal samples (e.g. Miszkiewicz and Mahoney, 2016; Burr et al., 1990; Pitfield et al., 2019a). While not all these applications are shared with biomedical approaches, we found that fundamental biological aspects of cortical bone histology, such as describing bone histological variation in different bones, with sex, and age (Caccia et al., 2016; Cooke et al., 2022; Cummaudo et al., 2018) can be directly comparable with samples obtained from autopsies or modern human donors (e.g. Thompson and Gunness-Hey, 1981; Burr et al., 1990), enriching the biomedical and forensic database (e.g. Gocha and Agnew, 2016; Maggio and Franklin, 2021; Pazzaglia et al., 2013; Matsuo et al., 2019). Another factor commonly investigated in biomedicine but also frequently found in bioarchaeological research related to relationships between secondary osteon formation and morphology and biomechanical load or physical activity and labour (e.g. Miszkiewicz, 2016; Miszkiewicz and Mahoney, 2016; Miszkiewicz et al., 2020; Pfeiffer et al., 2006; Pfeiffer, 1998; Raguin and Drapeau, 2020; Walker et al., 2023). While archaeological samples cannot yield experimentally derived measures of stress, strain, and load the way fresh bone can (such as through 3-point bend or compression testing, e.g. Singh et al., 2019), bioarchaeologists have linked secondary osteon densities and their geometric properties to measures of bone modelling reconstructed from cross-sectional geometry of long bones and anatomical regions that receive different mechanical stimulation (e.g. Miszkiewicz, 2016). These types of studies directly contribute to biomechanical research efforts within biomedicine as they explore spatial and anatomical macro-microscopic variation that might otherwise not be straightforward to measure in clinical samples unless segments of bone can be extracted post-mortem (such as the Melbourne Femur Research Collection, see Andronowski et al., 2025 for overview).

In the articles returned in our search, secondary osteon densities appear to be the only parameter that has been examined in cortical bone histology in fossils but is less often quantified than in archaeological contexts perhaps owing to limitations around sample sizes in palaeontology. Fossils from a single palaeontological locality might only preserve a few identifiable bones per species, from which permission to sample is further restricted. This is particularly the case for large and charismatic groups such as dinosaurs. However, some depositional contexts can preserve hundreds of thousands of bones representing hundreds to thousands of individuals, such as owl roost deposits, from which dozens of bones may be sampled (e.g. Louys et al., 2023). Bioarchaeological assemblages more often tend to preserve hundreds of skeletons in historical cemeteries, allowing for larger analyses and population level inferences. Thus, our review found that palaeontologists reporting bone histology often undertook qualitative descriptions related to the presence and absence of larger or fewer numbers of osteons (both primary and secondary), and made inferences about an animal's palaeobiology or used these to resolve phylogenetic questions (e.g. de Andrade and Sayão, 2014; de Farias et al., 2024; Downs and Donoghue, 2009; Martinez-Maza et al., 2014; Sombathy et al., 2025). From this perspective, and compared to bioarchaeological research, cortical bone histology findings from fossil animals are less relevant to biomedical research, although they still expand our knowledge of cortical bone histological variation in mammals (and other vertebrate classes such as reptiles, e.g. Sayão et al., 2016). These also provide positive contributions to understanding biomechanics. Two of the fossil studies we identified discussed analyses of osteon geometric properties, but these related to primary rather than secondary osteons in therocephalians (mammal-like reptiles) from South Africa (Huttenlocker and Botha-Brink, 2014) and a dyrosaurid (a crocodyliform) from Brazil (de Andrade and Sayão, 2014).

The next series of secondary osteon parameters included osteocyte lacunar densities, osteon circularity measures and lamellar wall thickness, although these form only a small proportion of our entire results history (Table 2). Cummaudo et al. (2018) and Miszkiewicz (2016) used osteocyte lacunae from across secondary osteons in archaeological human bone to explore their variation with age and sex, and whether this measure can be used for discrimination of species (human vs. pig specifically). While the study by Cummaudo et al. (2018) had forensic and bioarchaeological implications for distinguishing humans in assemblages of fragmented bone (finding minimal differences between humans and pigs), investigating inter-specific differences in lacunar densities within osteons also helps us better understand the osteoblast-osteocyte conversion during BMU activity applicable in biomedical contexts (e.g. Power et al., 2002; Qiu et al., 2005; Skedros et al., 2011a). Miszkiewicz (2016), using archaeological samples, reported a positive correlation between osteocyte lacunar density and cortical thickness of the posterior femur. She contextualised this finding in a biomechanical framework, furthering our understanding of remodelling responses along the linea aspera region of the human femur and thus contributing to osteocyte mechanosensing research in biomedicine (Voide et al., 2011; Hedgecock et al., 2007).

At least two bioarchaeological studies in our search have reported the use of secondary osteon circularity to investigate species discrimination based on bone histology (deer and humans in Lagacé et al., 2020), and age estimation (Karydi et al., 2022). There has been some discussion in the biomedical literature regarding the symmetry of secondary osteons as reflecting BMU activity (Smith et al., 2012; Wittig et al., 2019; Hegarty-Cremer et al., 2024), so insights from archaeological specimens can help us better understand that osteon circularity varies minimally between humans and other animals (Lagacé et al., 2020), and that it might have strong population-specific underpinnings (Karydi et al., 2022).

Secondary osteon wall thickness is surprisingly poorly represented in the sample of the articles we obtained, with only Burr et al. (1990) examining this variable in their analysis of human remains of 14th–19th century Pecos Indians from New Mexico (USA). In femoral samples, Burr et al. (1990) reported greater osteon wall thickness in the archaeological population compared to modern populations, but overall, there were no striking differences in bone health. This study contributed to a better understanding of population specific variation in bone histology, showing that the measure of osteon wall thickness itself holds a lot of potential for future archaeological cortical bone histology research as it reflects resorption activity (determined from resorption space contributing to lamellar wall thickness) and can also reflect net bone formation when measured statically (Ardizzoni, 2001; Skedros et al., 2011a).

None of the articles using ancient samples that we identified mentioned an analysis of collagen fibre orientation within secondary osteons, even though this has been a popular topic within experimental research (e.g. Skedros et al., 2006, Skedros et al., 2009, Skedros et al., 2011a, Skedros et al., 2011b, Skedros et al., 2011c, Skedros et al., 2011d, Skedros et al., 2024; Skedros and Doutré, 2019). One possible explanation for this limited engagement within bioarchaeological and palaeontological research could be the effects of taphonomic alteration on collagen preservation. Although vascularity may be preserved and remain visible in bone with extreme degradation as a formation of the mineral matrix of bone, the same cannot be said for the organic nature of collagen. Skeletal material from environments with poor organic preservation, such as tropical regions, may only exhibit small areas of collagen preservation within cortical bone, which palaeohistologists tend to find as evident using birefringence under polarised light (Villagran et al., 2017). These small pockets of preserved collagen, if present at all in samples with extreme taphonomic alteration, may not be deemed adequate for evaluation of collagen fibre orientation. However, birefringence may not be an appropriate method for studying collagen fibre orientation in bone since minerals can also cause birefringence and not be aligned in the same way as collagen fibres (Takano et al., 1996; Georgiadis et al., 2016). This highlights an area that is open to investigation, particularly in human archaeological specimens where there are large sample sizes of adequate preservation and could shed light on the distribution of different collagen fibre morphotypes, and possibly through an ultra-structural analysis accounting for hydroxyapatite crystallite orientation (Turner-Walker, 2019), within different bones and regions corresponding to tension or compression forces.

Likewise, bone formation rate and activation frequency as inferred from fluorescent labels within lamellae of secondary osteons have not been mentioned in the articles examining ancient samples we retrieved. Our search did return one article that reported data for bone formation rates and osteon activation frequencies using static histomorphometry methods. Using archaeological human ribs, Mulhern (2000) calculated secondary osteon mean activation frequency, defined as the mean number of secondary osteons generated per year per mm^2^, where accumulated osteon creations are divided by chronological age and the effective mean age of the adult compacta is deducted (based on Frost and Wu, 1967). While this is not the same as activation frequency studied using dynamic bone histomorphometry in fresh bone, it demonstrates an avenue for reconstructions of bone remodelling dynamics in ancient samples, and thus potential for comparability between biomedical and bioarchaeological disciplines. Although not retrieved in our PubMed analysis, Abbott et al. (1996; and see Streeter et al., 2010 for updated osteon calculations), also calculated bone formation rate and activation frequency using femur and tibia histology samples to compare the dynamics of bone remodelling across archaic and early modern humans from the Middle and Late Pleistocene. We also identified a palaeontological article for the formation rate parameter by Sombathy et al. (2025), but it refers to bone growth rates reconstructed from long bones of a Late Jurassic *Ceratosaurus* using lines of arrested growth, thought to represent a known time period, seen in thin sections rather than the quantification of secondary osteons.

While dynamic bone histomorphometry in the modern sense, where labels are injected in vivo, cannot be undertaken on ancient samples, there have been remarkable cases of bone histology samples from archaeological humans reported to incorporate tetracycline into bone tissue prior to death (Armelagos et al., 2001; Bassett et al., 1980). In an article by Bassett et al. (1980), which was not retrieved in our analysis, human femur bone histology from Sudanese Nubia dated to 350–550 CE viewed under a fluorescent microscope exhibited fluorescence seen in experimentally tetracycline-labelled bone tissue. Even though this sample predated the antibiotic era, Bassett et al. (1980) demonstrated that ancient Nubians might have consumed mouldy grains (wheat, barley, millet) that would have been stored in mud bins contaminated with tetracycline-producing *Streptomycetes*. This research has offered biomedical scientists an ancient perspective on tetracycline incorporation into forming secondary osteons.

One major limitation in all the articles identified in Table 3, Table 4 will always remain irreconcilably different from biomedical efforts, which is having to work with unknown demographic parameters. Chronological age is not known in ancient samples, so researchers estimate age-at-death from known age progressive skeletal changes, such as closure of skull sutures, degree of dental wear of the permanent dentition, eruption of deciduous and permanent teeth (Buikstra and Ubelaker, 1994). This produces a range of ages that are categorised into broad ontogenetic bins, such as young, middle-aged, and old (Clark et al., 2023; Buikstra and Ubelaker, 1994). The inability to ascribe secondary osteon changes to specific ages means analyses on ancient specimens cannot be directly compared to those in biomedical studies, but we do think that examining general or relative patterns and trends (such as lower secondary osteon densities in younger, and higher densities in older individuals) and assessing their variability within contexts (such as climate, social status, environmental change) provides a reference point for understanding bone biological adaptation in the past.

The same is true for biological sex, which tends to be estimated based upon macroscopic methods that compare sex-specific skeletal characteristics with standards developed from documented skeletal collections (Buikstra and Ubelaker, 1994). While these can have high accuracy, such as the ‘Phenice method’ which looks for the ventral arc, concave subpubic area, and a sharp and narrow medial aspect of the ischiopubic ramus characteristic of females (MacLaughlin and Bruce, 1990; Klales, 2020), these estimates are still probability statements, which is not the case in biomedical research. Unless the sex of ancient samples can be validated using ancient DNA or proteomic analyses (Hendy, 2021; Peacock, 2025), biomedical benefits gained from these samples are still limited to general trends and patterns and subject to possible misclassification of secondary osteon data to sex groups.

### 3.2 Bone type and research design

While almost all major bones have been sectioned in bioarchaeology and palaeontology in the articles we identified, the most commonly examined skeletal element across both disciplines is the femur, followed by the rib (Table 3, Table 4). The popularity of the femur for histological examination in ancient specimens mirrors its use within the biomedical literature (e.g. Pazzaglia et al., 2015; Watanabe et al., 1998; Yamada et al., 2013). Technically speaking, this likely relates to cortical bone sections removed from the femoral midshaft that offers a wide field of view where large quantities of secondary osteons can be easily identified and visualised. Conceptually, the femur is a major long bone involved in mobility and behaviour in most tetrapods, allowing testing of various biomechanical hypotheses with bone remodelling. The common justification in bioarchaeological and palaeontological papers for selecting the rib relates to the evaluation of bone remodelling rates that could be minimally impacted by significant mechanical load, and thus indicating factors such as diet (Pfeiffer et al., 2019). This has foundations in modern bone research examining intra-skeletal load variation and serves as a useful parallel for rib and the femur comparisons across disciplines (Skedros et al., 2013). However, this biomechanical perspective is easier to address with documented modern samples as interpretations using ancient specimens can be problematic (Pfeiffer et al., 2006). Pfeiffer et al. (2006) demonstrated that populations with behaviours that should correspond to larger or smaller secondary osteon and Haversian canal dimensions show inconsistent or opposite patterns than expected, making them complicated behavioural indicators.

Both bioarchaeological and palaeontological research we identified tend to section multiple different bones from one individual to describe histological variation. This is typically presented as a case study (Cummaudo et al., 2018), but can also involve larger sample sizes, particularly in palaeontology where a series of individuals are sectioned for ontogenetic comparisons (de Farias et al., 2024). This has great potential for mapping secondary osteon distribution with different bone size, type, and bone tissue proportions that would otherwise be difficult to arrange in clinical contexts or autopsies. For example, our search identified an article by Cummaudo et al. (2018) who sectioned the humerus, ulna, radius, clavicle, femur, tibia, fibula, metacarpal, metatarsal, glabella, frontal bone, parietal, occipital, temporal bone, scapula, sternum, rib, iliac crest, os coxa, sesamoid, patella, mandible, and cervical vertebra in an individual from Medieval San Martino di Serravalle's Church (Italy) to report that long bones showed a higher histological variability than flat and irregular bones that were more uniform, as well as cases of “osteon banding” (pattern of linearly arranged secondary osteons) and drifting osteons. This sort of insight is critical to biomedical research that might have surgical or healing goals for understanding how secondary osteons are distributed within the human skeletal systems.

Within palaeontology, it is also worth highlighting the histological study of osteoderms which are bony deposits found in the skin of some reptiles, dinosaurs, and some mammals (Bergqvist et al., 2019; Ebel et al., 2025; Ponce et al., 2023; Sombathy et al., 2025). While these do not play a major part of biomedical research, they carry significant evolutionary implications for understating the tetrapod transition from an aquatic to a terrestrial lifestyle (Ebel et al., 2025). Understanding their structure and function can also contribute to veterinary science and basic bone compositional research by exploring osteoderm bone matrix formation and calcification. In the articles identified, we have noted studies that described osteon densities of osteoderms in armadillos [*Riostegotherium yanei* and *Dasypus novemcinctus* (Bergqvist et al., 2019)]; archosaurs [*Fasolasuchus tenax* and *Prestosuchus chiniquensis* (Ponce et al., 2023)], and non-avian theropod dinosaurs [*Ceratosaurus* (Sombathy et al., 2025)], all noting that the internal organisation of osteoderms might be species specific and thus reflective of the environmental and intrinsic biological factors.

### 3.3 Technical remarks

Histological procedures implemented to make bone thin sections both in archaeological and fossil samples are almost the same as in biomedical research, except samples in the former are undecalcified and unstained, and epoxy resin embedding (rather than methylmethacrylate embedding) is more common. All the papers we identified cut embedded samples on a low-speed saw, grind, polish, dehydrate, clear and (mostly) coverslip the thin sections and then examine them using polarised and transmitted light, capturing images using microscope high resolution cameras. There is also no need for tissue fixing or dehydration prior to embedding since the specimens are dry. This means that archaeological and fossil samples could be easily made into thin sections in biomedical laboratories, facilitating collaboration and access to large sample sizes. This would not work the other way as in most cases histological laboratories in bioarchaeology and palaeontology units are set up like petrographic geological laboratories and might not have appropriate anatomy licences for storing and handling modern human tissues.

It is remarkable that the level of histological preservation in some ancient bone samples can be comparable to modern samples (Fig. 1), and that this alone provides an opportunity for collaboration between biomedical and bioarchaeological or palaeontological researchers, since all fields are essentially looking at the same biological microstructure. Having said this, pristine preservation at the histological level in bioarchaeology and/or palaeontology is still rare. This can lead to localised, or widespread, artifacts seen histologically that do not originate from bone biology and that can obscure, if not modify, histological features. Microcracks are often seen in ancient bone samples (that differ from microdamage relating to mechanical load) (Nacarino-Meneses et al., 2021; Thompson et al., 2024), and bone tissue can be discoloured (Thompson et al., 2024; Jans, 2021). The degradation can be so intense that an entire bone sample is unusable. Further, there can be issues with cortical porosity that might be due to microbial tunnelling or mineral dissolution, which is not the case in fresh bone (Turner-Walker, 2023). This limits the one other obvious medical area that archaeological bone histology samples can contribute to, which is around bone metabolic diseases such as osteopenia and osteoporosis. Although this is harder to decipher in cortical bone in early stages of osteoporosis (advanced osteoporosis can lead to cortical bone trabecularisation), rather than trabecular bone, and certainly cannot be diagnosed based on histology alone, attempts to link possible osteoporosis experiences from advanced cortical bone porosity interpreted as prolonged resorption by osteoclasts in archaeological samples have to be sure that that these are not due to preservation issues (Miszkiewicz et al., 2022). Researchers in this space have been implementing the Oxford Histological Index (OHI) which allows for an assessment of bone preservation on a relative scale and to identification of samples that are comparable to modern bone (category 5 of the OHI) (Hedges et al., 1995). Further, osteocyte lacunae from ancient samples do not house osteocytes, while fresh samples allow for the study of osteocytes as well as osteoblasts and osteoclasts. However, a good proxy for osteocytes in ancient samples is the lacunae themselves, but these too can be altered due to post-mortem degradation.

### 3.4 Identifying avenues for interdisciplinary complementary research

Based on our review of the articles identified in our PubMed search, we provide a list of specific bone biology areas that can benefit from interdisciplinary collaboration with bioarchaeology and palaeontology. The direction of our suggestions is from the ancient samples to contributions to biomedical research, but this is somewhat an inherently two-way relationship as palaeontologists and bioarchaeologists rely on modern research to interpret bone microstructure in ancient samples. Table 5 lists those areas along with broader research questions and suggested ancient bone materials for addressing these questions. Although a further discussion is beyond the scope of our study, we do want to highlight that, similarly to biomedical research, there are important ethical considerations when working with archaeological and palaeontological materials. As histology is an invasive method, different levels of approvals for removing samples are often required, and these range depending on the curating institution, descendant communities involved, and with other local national regulations.

**Table 5:** Biomedical bone biology areas (in no particular order) that can benefit from interdisciplinary collaboration with bioarchaeologists and palaeontologists, or by using ancient bone materials.

Research area with (sub)discipline(s) in brackets	Broad questions	Ancient bone materials complementing modern samples
Bone adaptation to biomechanical load (biomechanics, bone biology, orthopaedic surgery and engineering)	Secondary osteon distribution with different anatomical bone regions and bone gracileness/robusticity Bone remodelling variation with physical activity/labour/mobility	Archaeological and fossil long bone cross-sections, spanning humans and other animals, where macroscopically measured robusticity can be assessed and examined against secondary osteon densities and geometric properties, and behavioural context
Bone growth and development during early ontogeny and with age throughout adulthood (developmental biology, bone biology)	Bone histology change with age from infant to young adulthood stages Quantification of secondary osteon densities at different adult age stages	Archaeological and fossil human and other animal samples from various bones sampling age series Larger sample size could be obtained in more recent human archaeological collections (such as from medieval cemeteries), but it would usually be smaller in palaeontological collections
Intra-skeletal bone microscopic variation (anatomy, bone biology)	Bone histology variation with bone type (size, shape, development timing, mechanical utility) within one individual	Archaeological and fossil human and other animal specimens where an entire skeleton (or at least all the major bones) is preserved can undergo sampling of all the bones of interest to quantity bone remodelling and other secondary osteon properties within the same individual
Microscopic structure and composition of mineralised tissues (materials science, structural biology, bone biology)	Bone histology tissue proportions and distribution in relation mineral content	Archaeological and fossil human and other animal bone samples, including osteoderms Even a bone/osteoderm fragment with limited context can be useful here
Genetic underpinning to bone health and strength (genetics, molecular biology, bone biology, human variation)	Bone histology/remodelling differences between genetically distinct groups	Archaeological human bones originating from collections characterised genetically (through ancient DNA)
Evaluation of different mammalian models as proxies for understanding bone metabolic disease in humans (comparative anatomy, translational medicine, animal science, bone biology)	Bone histology/remodelling differences between different animal species and taxa, and their comparison with humans	Archaeological and fossil animal bones that undergo remodelling and experience the formation of secondary osteons when alive (e.g. larger mammals rather than rodents)
Influence of diet on bone remodelling (nutritional science, endocrinology, public health, bone biology)	Bone remodelling variation/change with nutritional information	Archaeological and fossil animal bones that have had dietary proxies established, such as protein consumption through nitrogen stable isotope analysis of bone collagen
Histological manifestation of bone diseases (pathophysiology, bone biology)	Bone remodelling disruption due to disease Bone histology tissue morphological manifestation in various disease states	Archaeological and fossil animal bones with macroscopically diagnosed skeletal conditions (or primarily non-skeletal conditions that lead to skeletal manifestation)
Social determinants of bone health (public health, epidemiology, bone biology)	Bone remodelling differences between groups of different socio-economic (SES) backgrounds	Archaeological human bone samples from regions and historical time periods affected by intense SES stratification, such as in the medieval period and during the Bronze Age
Evolution of bone remodelling/biology (functional morphology, comparative anatomy, bone biology)	Bone remodelling differences between various taxa and species from distinct geological time periods Bone remodelling differences between human groups dated to various time periods	Archaeological and fossil animal bones with relative dates (specific year is not possible to reconstruct, but broad comparisons between different time periods can be undertaken)

### 3.5 Limitations and disciplinary categorisation

We recognise that there are more histological papers that have dealt with archaeological and palaeontological samples (as well as other biomedical research) than what our output yielded (e.g. Padian et al., 2016). A large portion of those includes investigations into disease, broadly termed palaeohistopathology (Schultz, 2001), which relies heavily on morphological descriptions of atypical bone microstructure with limited use of standard bone histomorphometry (Cooke, 2024; de Boer et al., 2013). Our study is not meant to be exhaustive but to provide a sample of larger literature showing a representative proportion of interdisciplinary research as captured by PubMed. We feel that interdisciplinary variation has been captured by our search in considering nine histological parameters, although our results are clearly biased by whether or not a journal is indexed in PubMed.

We also extracted articles that specifically listed the use of archaeological or fossil samples, which was determined based on the age/date of the material. However, three other studies were identified that do not directly assess archaeological or fossil materials but have aims that are specifically targeting those disciplines. Heck and Woodward (2021) described bone histology of femora, tibiotarsi, tarsometatarsi, humeri, ulnae and radii in modern individuals of a 14-year-old male and 5-year-old female North Island Brown Kiwi (*Apteryx mantelli*) with the purpose of providing a comparative database of modern taxa for extinct vertebrate growth and life history. This example shows that palaeontologists also work with modern samples, indirectly contributing to bone biology that could have biomedical implications while informing inferences made from fossils. There are also researchers who work with historical collections of thin sections, such Felder et al. (2017) whose paper examined allometry between body mass and secondary osteon size in the humerus and femur from a range of mammals. Felder et al. (2017) worked with a 19th century collection of microscope slides prepared by John Thomas Quekett, which is now known as one of the oldest museum collections of bone histology and forms part of collections at the Royal College of Surgeons (Quekett, 1855). While this collection is neither archaeological nor palaeontological, it does have a historical background and shows immense potential in biological analyses that have implications for biomedical and fossil research, in addition to demonstrating that histological slides can be curated and preserved for multiple generations of researchers. A final example of an interdisciplinary paper that did not quite meet our inclusionary criteria is the study by Beresheim et al. (2020) that examined human rib tissue properties in an age series (birth to 21 years) from the 1729–1857 Spitalfields cemetery in London. This sample is archaeological, but it comes with an added quality of having known ages at death from preserved coffin plates which is typically not the case for populations from earlier time periods. In this paper, Beresheim et al. (2020) undertake microCT analyses of the rib, results from which are then compared to prior bone histology work on the same sample, finding, for example, that porosity in cortical bone measured three dimensionally does not correlate with two-dimensional osteon densities. This is an example of a study using archaeological samples that would be difficult to obtain in modern contexts due to the age series needed, and thus adds to biomedical knowledge.

## 4 Conclusions

Secondary osteon examination using histomorphological and histomorphometric methods is clearly a versatile tool that has applications in bone studies across a range of disciplines. Its examination in samples from archaeological and fossil specimens has potential to contribute to current biomedical and anatomical efforts in understanding bone structure and its metabolic activity as it relates to various factors such as age, sex, and behaviour. We found that approximately 9 % of peer reviewed articles reporting new secondary osteon data indexed by PubMed represent archaeological or fossil material. We highlight that secondary osteon static histomorphometry can be applied to modern and archaeological human, and extinct and extant animal samples, yielding comparable data for parameters such as osteon population density and geometric properties of secondary osteons and Haversian canals. This methodological avenue offers potential for evolutionary and temporal exploration of bone health and microanatomical structure, providing time depth to our understanding of bone biology. We do note that modern bone histology reflects only biology, whereas archaeological and palaeontological bone histology reflects both biology and post-mortem and diagenetic processes. While the underlying bone microstructure is the same across all these disciplines, ancient samples face a challenge that is absent in medicine: disentangling post-mortem from biological signals. Nevertheless, our review highlights an underutilised collaboration between palaeohistology and biomedicine that has potential to improve our current understanding of the nature of secondary osteons and remodelling within cortical bone.

## CRediT authorship contribution statement

**Justyna J. Miszkiewicz:** Writing – review & editing, Writing – original draft, Validation, Project administration, Methodology, Investigation, Funding acquisition, Formal analysis, Data curation, Conceptualization. **Karen M. Cooke:** Writing – review & editing, Methodology, Investigation, Formal analysis. **Holly E. Reid:** Writing – review & editing, Investigation, Formal analysis. **Julien Louys:** Writing – review & editing, Methodology, Investigation, Formal analysis.

## Informed consent and ethics approval

Not applicable.

## Funding

This work was supported by the 10.13039/501100000923Australian Research Council [FT240100030].

## Declaration of competing interest

None to declare.

## Data Availability

Provided as Supplementary Material.

## Appendix A Supplementary data

Supplementary material Image 1
